# Knowledge, Attitudes, and Practices in Relation to Mosquito-Borne Diseases in Bangladesh

**DOI:** 10.3390/ijerph19148258

**Published:** 2022-07-06

**Authors:** Mir Mobin, Mohammad Khan, Hasnain Anjum, Habibur Rahman, Mahfuza Marzan, Md Asiful Islam

**Affiliations:** 1 Department of Microbiology, Jahangirnagar University, Savar, Dhaka 1342, Bangladesh; mirmubin2528@gmail.com (M.M.); habiburju46@gmail.com (H.R.); 2 School of Dental Sciences, Universiti Sains Malaysia, Kubang Kerian 16150, Malaysia; drmohammadkhan1001@gmail.com; 3 Department of Microbiology, Primeasia University, 12 Kemal Ataturk Ave, Dhaka 1213, Bangladesh; hasnain.anjum@primeasia.edu.bd; 4 Department of Chemistry, University of South Florida, 4202 E Fowler Ave, Tampa, FL 66320, USA; 5 Department of Haematology, School of Medical Sciences, Universiti Sains Malaysia, Kubang Kerian 16150, Malaysia; 6 Institute of Metabolism and Systems Research, University of Birmingham, Birmingham B15 2TT, UK

**Keywords:** mosquito-borne diseases, knowledge, attitude, practice, Bangladesh

## Abstract

Mosquito-borne diseases (MBDs) such as dengue, malaria, and chikungunya are common in Bangladesh, with frequent outbreaks in the rainy season. Analysis of the knowledge, attitudes, and practices of people toward any crisis is fundamental to addressing any gap. Here, we conducted a cross-sectional study mainly focusing on the northern, southern and central parts of Bangladesh to understand the level of knowledge, attitudes, and practices of people regarding MBDs, mosquito habitats, or control measures. A total of 1720 participants were involved in the study from 33 out of 64 districts of Bangladesh, of which 56.9% were male. While most of them knew about dengue (97.1%), chikungunya (81.4%), and malaria (85.2%), only half of them were aware of filaria (53.3%), which is endemic to the northern region. A knowledge score (0–8, low), (9–16, moderate), (17–24, high), and attitude score (0–4, poor), (5–8, moderate), and (9–13, high) were assigned. While poor and moderate attitudes were considered negative, good attitudes were considered positive. About 45% of the respondents had a moderate knowledge score (50–70); however, about 67.9% of participants showed a good attitude score (>70) towards the control of MBDs. It was found that the knowledge and attitude of the responders were related to their profession (knowledge *p* < 0.001; attitude, *p* = 0.002), residential area (knowledge *p* < 0.001; attitude, *p* < 0.001), and education level (knowledge *p* < 0.001; attitude *p* = 0.004). A mosquito is a kind of nuisance bug, and about 79.8% of responders admitted that they kill mosquitoes as soon as they notice them. They also use bed nets (93.7%) followed by mosquito coils (85.7%) as a preventive method. Interestingly, 73.2% of the responders were reluctant to contact the local government during an increase in mosquito numbers. Overall, the people of Bangladesh have a positive attitude towards the prevention of mosquito-borne diseases. It is highly recommended that the government creates more knowledge regarding this issue and develops collaborative approaches with local people to implement robust preventive measures against mosquito-borne diseases.

## 1. Introduction

The mosquito is the world’s deadliest creature. They caused around 750,000 deaths worldwide through the transmission of mosquito-borne diseases (MBDs) in 2018 [1,2]. An MBD occurs when mosquitoes infected with parasites or viruses transmit the pathogens to their host while feeding on the host’s blood. The most common MBD is malaria, with the highest fatality rate and the highest (65,493.1) disability-adjusted life years (DALYs), followed by lymphatic filariasis (DALYs 2022.1) and dengue (DALYs 1142.7) [3]. Around 2400 million people worldwide are at risk of developing malaria, whereas dengue can cause 100–400 million estimated infections each year [4,5]. In addition to health suffering and death, economic losses due to MBDs are underrated. The direct cost of MBDs for treatment and prevention purposes spent by the government and individuals, as well as indirect costs due to productive labor time lost, is not accumulated and thus overlooked. A study estimated that the annual cost of dengue illness in the Americas is USD 2.1 billion, without the cost of preventive measures [6]. However, this can create a huge economic burden for developing countries such as Bangladesh. A recent study showed that the aggregate economic expenditure for dengue treatment in Bangladesh is around USD 15.27 million a year [7].

Bangladesh is prone to MBDs due to the combination of several factors such as climate change, rapid urbanization, increased human mobility, improper drainage systems, illiteracy, and lack of awareness [8,9]. About 33.6% of the population of Bangladesh is at risk of contracting malaria, while it is estimated that around 70 million people are at risk of developing lymphatic filariasis [10]. Bangladesh saw a massive outbreak of dengue fever in 2019, where 100,000 people were infected with 197 confirmed deaths, and an outbreak of chikungunya fever in 2017, where more than 13,000 were infected [11,12]. A recent study reported the high prevalence of Japanese encephalitis in Bangladesh, as around 8% of patients with meningitis-encephalitis syndrome were diagnosed with it [13]. However, due to a lack of proper surveillance and reporting, we can assume the number to be higher than reported. The increase in global movement and presence of appropriate vectors has made the introduction of other MBDs such as Zika, West Nile fever, and yellow fever into Bangladesh a matter of time. Remarkably, different regions of Bangladesh showed variations in the endemicity of different MBDs. The southern hill region is malaria-endemic, while most lymphatic filariasis cases are reported in the northern part of the country [14]. Though dengue and chikungunya cases were documented all around the country, the highest prevalence was present in the central part of the country due to urbanization and dense population, which favors the breeding of its vector, *Aedes* spp.

Current control strategies of MBDs largely rely on vector control through the use of synthetic pesticides against mosquito larva or their mature form. However, it has negative impacts on the environment, high operational costs, and there is a chance of developing resistant mosquito populations [15,16]. Microbial controls of mosquitoes through the application of entomopathogenic fungus and symbiotic bacteria, genetic modification, and plant-derived, eco-friendly, insecticidal nanoparticles are gaining much attention [15,17]. Enormous efforts have been made toward the development of vaccines against some of these MBD pathogens, but with limited success [18,19].

The increase in mosquito-borne diseases is highly anthropogenic and connected to global climate change, involving high temperatures or rainfall that affects mosquito bionomics [20]. However, simple human intervention and behavioral changes can reduce the mosquito load significantly [21]. On the other hand, education and knowledge regarding MBD and mosquito control can lead to improved attitudes of citizens and help them to adopt protective practices [22]. Previous knowledge, attitude, and practice (KAP) studies conducted in different regions of Bangladesh on malaria and dengue have demonstrated a knowledge gap among community dwellers regarding the transmission, prevention, or treatment of MBDs and their reluctance to perform preventive measures [23,24]. Another study conducted among university students in Dhaka City, Bangladesh, showed good knowledge (66.72%), attitude (89.28%), and practices (68.32%) scores, though the authors emphasized the inclusion of infectious disease-related subjects into the university curriculum [25]. However, most of these studies were focused on a specific MBD or carried out in communities of a specific zone, such as hilly tracts or city areas. The aim of this study was to conduct a KAP survey on common MBDs (malaria, lymphatic filariasis, dengue, and chikungunya) in Bangladesh, involving people of all walks of life to understand different socio-economic predictors of improved knowledge, attitude, and practices (KAP) regarding MBDs.

## 2. Methods

### 2.1. Study Design, Location, and Sample Size

**Figure 1:**
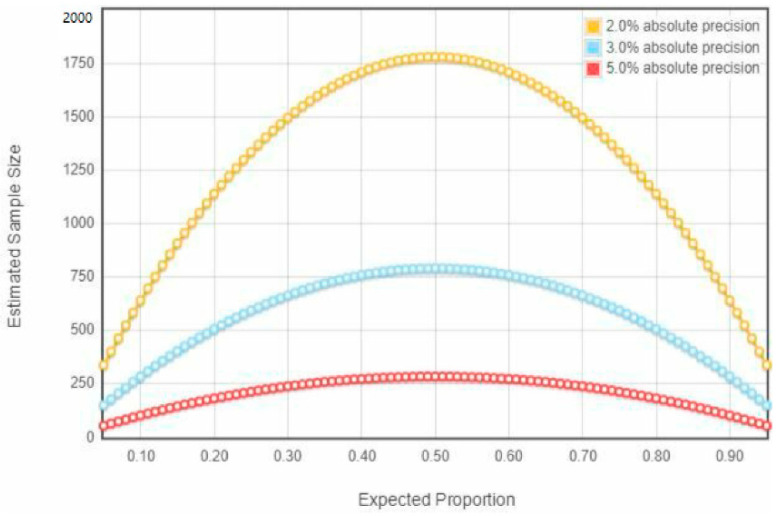
Sample size calculation.

A cross-sectional study was conducted with a structured questionnaire to understand the knowledge, attitude, and practice of MBDs. The study was performed between December 2020 and June 2021. The study sites included 33 out of 64 districts (administrative units) of Bangladesh where some MBDs are prevalent. The sample size was calculated using a single proportional formula. A total of 1750 study subjects were initially planned to be included for knowledge, attitudes, and practice assessment with the help of studies done in Karnataka [26] and El Salvador [27], where the expected precision margin was 0.05 with an expected response rate of 95% (Figure 1).

### 2.2. Development of Questionnaire, Distribution, and Data Collection

The questionnaire was primarily developed based on a review of the previously published literature with slight modifications to adjust for the cultural practices of Bangladesh [23,28]. Furthermore, to validate the questionnaire, a pilot study was conducted on 100 people. The face validity and content validity were carried out with the help of expert faculty, parasitologists, tropical disease specialists, public health experts, and epidemiologists. The language sustainability and easiness were confirmed with the help of the Department of Bangla language and literacy. The internal consistency was found to be 0.73 using Cronbach’s alpha. Following the analysis of the pilot study results (not included in the final results), some modifications were added to strengthen the questionnaire. The questionnaire has four sections. The first section is demographical data (7 questions), and the second, third, and fourth sections contain questions regarding knowledge (11 questions), attitude (10 questions), and practices (7 questions), respectively. The questionnaire was delivered by the data collectors and volunteers. Based on the availability of the data collectors, we only recruited 33 districts out of 64. Data were collected over 6 months with the help of 140 data collectors, who were also undergraduate students in the public health and microbiology departments. The data collectors and students were trained about the aim and objective of the study, the procedure to approach people to collect data, and data accuracy through a day-long workshop. In the case of tribal people, we involved a student from their community to ease the data collection. As it was a closed type of questionnaire, we did not encounter any non-response rate. However, there was selection bias and information bias, which was inevitable due to illiteracy and different dialects used across the country. Responses were marked in the printed questionnaire. After that, the data were uploaded directly to the SPSS software by the data collectors.

### 2.3. Data Analysis

The demographical characteristics of the participants were presented as percentage and frequency tabulations. Each participant was given a score in the knowledge and attitude section that ranged from 0 to 24 and 0 to 13, respectively. The scores were further divided into low/poor and moderate. For knowledge (0–8, low), (9–16, moderate), (17–24, high), and attitude (0–4, poor), (5–8, moderate), and (9–13, high). While poor and moderate attitudes were considered negative attitudes, good attitudes were considered positive. Questions related to knowledge and attitude were assessed using a three-point Likert scale, with the options “True”, “False”, “Uncertain”, and “Agree”, “Disagree”, and “Not sure” in respective sections. Logistic regression analysis was used to calculate crude and adjusted odds ratios. The predictors of high knowledge and attitude scores, as well as good practice behaviors, were also calculated.

## 3. Results

### 3.1. Sociodemographic Characteristics of the Population

**Figure 2:**
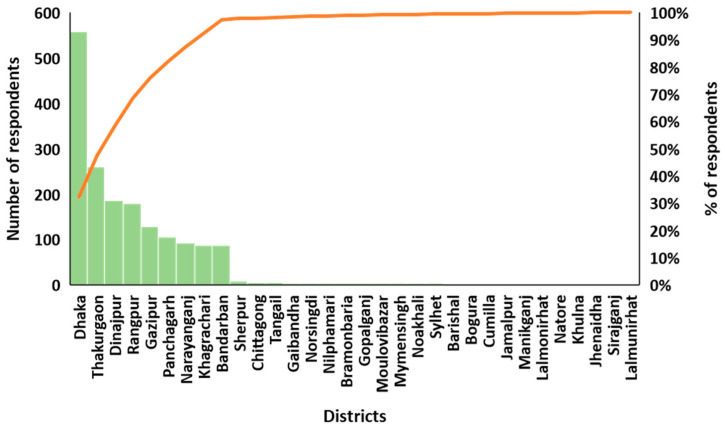
Respondents from different districts in Bangladesh.

**Figure 3:**
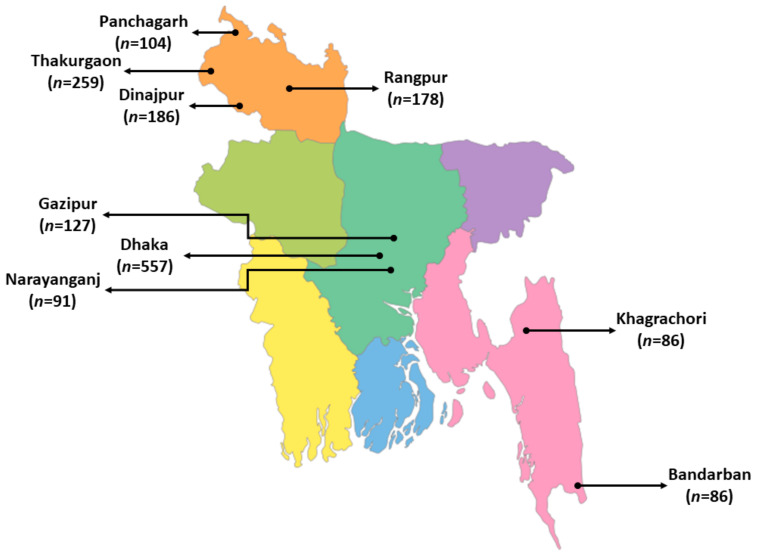
Geographical representation of the most frequent respondents from different regions in Bangladesh.

In total, 1720 individuals participated in this study from December 2020 to June 2021. Of these, 557 individuals were from the Dhaka district, followed by 259 from Thakurgaon, 186 from Dinajpur, and 127 from Rangpur (Figure 2 and Figure 3). Most of the respondents were in the young (18–28 years old) age group (52.2%) (Table 1). The majority of the respondents were male (56.9%), students (35.0%), or employed (28.0%). Among the respondents, 464 individuals were undergraduates (27.0%), followed by 16.6% graduates, while 13.8% had below SSC level education or no education at all (9.0%). Most of the respondents did not want to disclose their monthly income (42.3%), while 16.9% had a monthly income below BTD 10,000. Half of the respondents lived in urban areas (53.3%), 1456 individuals (84.7%) had TV, followed by internet access (66.9%).

### 3.2. Knowledge among the Respondents

Almost all the respondents had heard about MBDs (97.6%) (Table 2). Most respondents knew about dengue (97.1%), chikungunya (81.4%), and malaria (85.2%), while only half of them knew about filaria (53.3%). Regarding the item about vector bite times, 76.3% knew about dengue and chikungunya compared to malaria (58.9%). *Aedes* spp. was the most known mosquito among the respondents (90.9%), followed by *Anopheles* spp. (51.9%) and *Culex* spp. (34.7%). Most of the respondents were aware of mosquito breeding areas. Muscle pain (74.6%) was the most reported additional symptom for dengue, followed by elephantiasis (66.0%) for filariasis, vomiting (55.8%), and muscle pain (54.3%) for malaria. The overall mean and standard deviation (SD) of knowledge score among the respondents was 60.7 ± 18.1 (Table 3). Most of them had a moderate level of knowledge about MBDs (45.0%).

### 3.3. Attitudes

Overall attitude scores among the respondents were 80.4 ± 13.1 (Table 4). More than half of the respondents had good attitudes toward MBD eradication and prevention (67.9%). Amongst the respondents, most agreed that we can prevent MBDs (94.5), it is a problem for Bangladesh (97.3%), it can be fatal (93.3%), and anyone can be affected by this (96.9%) (Table 5). Almost all the respondents agreed that most awareness needs to be generated for prevention (97.1%). Additionally, 1645 individuals agreed that besides the government, all citizens should work for prevention (95.6%), while 659 (38.3%) disagreed that the government is taking enough steps for the prevention of MBDs. Unawareness (94.7%) and lack of government approach (48.4%) were identified as major obstacles to the prevention of MBDs. Nearly three-fourths of respondents believed that their surroundings have a breeding place for mosquitoes.

### 3.4. Factors Related to the Knowledge Score and Attitude Score

The linear regression analysis of knowledge score and sociodemographic factor showing that the profession of the respondents (β = −0.36; *t* = −17.223, *p* < 0.001), residential area (β = 0.24; *t* = 12.086, *p* < 0.001), and education level (β = 0.31; *t* = 15.81, *p* < 0.001) have significant influences on individual’s knowledge about mosquito-borne diseases (Table 6). The linear regression analysis of attitude scores and sociodemographic factors showing that the profession of the respondents (β = −0.08; *t* = −3.086, *p* = 0.002), residential area (β = 0.17; *t* = 7.020, *p* < 0.001), and education level (β = 0.07; *t* = 2.871, *p* = 0.004) have significant influences on individual’s attitude about mosquito-borne diseases (Table 7).

### 3.5. Prevention Practices against Mosquito-Borne Diseases

More than two-thirds of the respondents said they killed the mosquitoes as soon as they noticed them (79.8%) (Table 8). Bed nets (93.7%) were the most common preventive method used against mosquito bites, followed by coils (85.7%), electric bats (42.0%), and insecticide spray (33.1%). The majority of participants declared that they cleaned their surroundings (76.0%). Surprisingly, more than two-thirds of the respondents never contacted the local government if mosquitoes increased in their locality (73.2%). Half of the respondents were found to check and clean water storage in their houses (50.6%) and seek medical help if they feel feverish (50.0%).

## 4. Discussion

Mosquito-borne diseases are considered a major health threat in Southeast Asian countries such as Bangladesh because of unplanned and impromptu urbanization and overpopulation, which serves as a suitable environment for rapid dissemination of MBDs such as dengue, malaria, filariasis, and chikungunya [29]. To prevent and control such life-threatening diseases, socio-demographical factors as well as the KAP among the population play critical roles [30]. The study was conducted to assess the KAPs regarding MBDs and their prevention in different regions of Bangladesh.

Results obtained from the investigation showed evidence of rather good knowledge about major MBDs in the urban population of Bangladesh. The majority of the respondents were familiar with mosquito-borne diseases (98%), and among the diseases, dengue was the more commonly known (97%). Only half of the respondents were familiar with filariasis (53%), while malaria (85%) and chikungunya (81%) were relatively well-known. These findings can be compared with another study conducted by Saha et al. [31], where it was found that 89% of respondents were aware of malaria, while a recent study by Farzana et al. [32] reported that 97% of the population was familiar with dengue fever, supporting our data. The data can also be compared with similar studies conducted in Nepal [33], India [34], and Jamaica [35].

Knowledge regarding the nature of mosquitoes among the respondents revealed that the majority of the participants were familiar with the name *Aedes* spp. (91%), while half of them knew about *Anopheles* spp. (52%). The maximum number of respondents that were aware of the fact that dengue and chikungunya-carrying mosquitoes bite during daytime was 74%, which is similar to the findings of Rahman et al. [36]. Regarding the malarial vector, 59% of the respondents correctly answered that the biting period of malarial vectors was during nighttime, which is a higher number than found in a previous study by Saha et al. [31]. When asked about the breeding environment of mosquito vectors, about 86% of the participants replied that mosquitoes could breed in stagnant water, while 63% answered that mosquitoes could be born in both clean and dirty water. These data can be compared with a previous study by Abir et al., where most of the participants were aware of the fact that mosquitoes lay eggs in clear water [37]. However, a previous study by Dhar–Chowdhury et al. claimed a much lower percentage (87%) of awareness among residents of Dhaka City [38].

In regard to the knowledge about the symptoms of dengue fever, three-fourths of the respondents (75%) replied that muscle pain was the most common symptom alongside fever. Most of the respondents’ knowledge accuracy regarding other common symptoms of dengue fever was significant, including rash (58%), vomiting (57%), and pain behind the eyes (49%). However, a considerable number of participants did not know most of these common symptoms. This result can be compared with another similar study about dengue fever among university students in Bangladesh, where the accuracy of knowledge among respondents was much higher than in the current study (joint pains (92.5%), muscle pain (82.4%), and pain behind the eyes (70%)) [32]. However, this fact can be linked to the educational background of the respondents, as only 43.6% of respondents from the present study had an education of undergraduate level or above compared to the aforementioned study conducted on the university students. That study claimed a statistically significant relation between the KAP level of respondents and their academic attainment [32]. In the case of filariasis, major symptoms, along with fever, known to respondents were elephantiasis of different organs (66%), axillary lymphadenopathy (93%), and skin exfoliation (25%). When asked about symptoms of malarial fever, 56% of the participants said that vomiting was the major symptom of malaria besides fever, while 55% and 44% of the respondents acknowledged muscle pain and diarrhea to be other significant symptoms, respectively. This can be compared to a previous study by Saha et al. [31] about malarial symptoms, where 63% of the respondents knew the principal signs and symptoms of malaria accurately. The present study showed that the overall mean knowledge score among the respondents was 60.7 ± 18.1, which is satisfactory. Most of the participants had a moderate knowledge (45.0%) about mosquito-borne diseases, which is essential for the prevention of MBD outbreaks.

Regarding the attitudes toward mosquito-borne diseases, the majority of the participants of the present study responded positively, as about 97% of the respondents acknowledged the effects of MBDs. Overall, 68% of the participants had good attitudes towards mosquito-borne disease eradication and prevention. Most of the respondents in this study agreed that mosquito-borne diseases are a major problem in Bangladesh (97%) and can be fatal (93%), but they were also hopeful about the prevention of mosquito-borne diseases (95%). These data support a recent study conducted in Bangladesh claiming that 85% of the population agreed that MBDs such as dengue could be prevented [32]. However, in a similar study conducted in Western Australia, people showed much less concern about MBDs as only 25% of the respondents acknowledged mosquitoes as a health risk, while a major portion of them (43%) considered the bug as a nuisance [39].

When asked about awareness among general people, 97% of the respondents agreed that more awareness should be generated for effective prevention, and 79% replied that they were aware of the mosquitoes breeding in their surroundings. On the subject of the role of government in the prevention and eradication of mosquito-borne diseases in Bangladesh, 46% of the participants responded positively, as they felt the government is taking enough steps to prevent mosquito-borne diseases, but 39% were dissatisfied with the involvement of government in the matter. When opinions about the role of citizens were inquired about, most of the participants (97%) strongly agreed that an active role of the citizen is required in addition to the government to prevent mosquito-borne diseases. More than half of the respondents (64%) fear that there is a chance of new types of MBDs emerging in Bangladesh.

The majority of the respondents (95%) considered “unawareness” as the major obstacle in preventing MBDs, reflecting the fact that a major proportion of the population is still ignorant of information regarding MBDs, such as mosquitoes, their breeding habitats, signs and symptoms related to different kinds of MBDs, and preventive measures. Other factors deemed by participants as obstacles in MBD prevention included negative public attachment (53%), less effective role of government (49%), and financial constraints (34%). Though most of the respondents demonstrated positive attitudes toward MBD control and prevention, they also indicated insufficient measures taken by the government in this regard. However, a majority of respondents also acknowledged the impact of their individual actions on the community in the prevention and control of MBDs. In fact, in countries like Bangladesh, it is quite impossible for the government to control MBDs alone. Similar studies conducted in Western Australia and Congo noted a stark difference, however, citing the local participants’ attitude towards community, as they recognize their duties only for self-protection for their own household, but not for local communities [40,41].

On inquiring about practices among respondents to prevent MBDs, bed nets were found to be the most common. Of the participants, 94% were found to use bed nets regularly to prevent mosquito bites, while other popular preventive measures included mosquito coils (86%), fans (45%), electric bats (42%), and insecticide sprays (33%). These practices are common across the region, as similar practices were noted in several previous studies conducted in Bangladesh [31], India [42], and Jamaica [35]. About three-fourths of the participants claimed that they clean their surroundings, but only half of the participants were practicing regular cleaning of their household water storage, reflecting a gap in practice for proper control of mosquito breeding.

Interestingly, a large portion of the respondents (73%) were reluctant to contact the local government in case of increased mosquito infestations, indicating a communication gap between the local government and the people. In contrast, the Western Australian people mentioned in a previous study were more trusting of their government’s actions [41], which in turn provided the decision-makers an opportunity to get closer to the community and for healthcare personnel to be involved and maximize the educational activities among the population. In Bangladesh, the government could achieve a similar level of involvement with the people by proper and effective utilization of media, including the internet and visual platforms, to encourage a dependable relationship between government and local communities to successfully control and prevent mosquito-borne diseases. Social and environmental awareness and responsibilities should also be acknowledged for an effective prevention regime.

Although we collected a large sample in our current study, it does not properly represent the whole country due to the skewness of the responses from big cities. It is important to point out as a weakness of this study the possibility of bias in the sampling. Despite our best efforts to obtain a representative sample of survey respondents, we completely relied on our volunteers and students from Jahangirnagar University and a local research team, who collected data from their hometown or residing town. Our questionnaire was a closed type, which helped us avoid non-response bias. If a respondent did not understand the question, the person collecting the data told him or her to try again. People often do not practice what they preach. Their attitude might be different when the researcher is away. The results should be taken cautiously with more constructive measures.

## 5. Conclusions

Overall, the people of Bangladesh have a positive attitude towards the prevention of mosquito-borne diseases. It is highly recommended that the government creates more knowledge regarding this issue and develops collaborative approaches with local people to implement robust preventive measures against mosquito-borne diseases.

## Figures and Tables

**Table 1:** Sociodemographic characteristics of the respondents.

Variables	Frequency (%)
**Age (years)**	
18–28	899 (52.2)
29–38	334 (19.4)
39–48	252 (14.7)
49–58	157 (9.1)
58+	78 (4.5)
**Gender**	
Male	980 (56.9)
Female	729 (42.4)
Prefer not to say	11 (0.6)
**Professions**	
Employed (GO *, NGO, Private)	482 (28.0)
Student	602 (35.0)
Businessman	126 (7.3)
Housewife	329 (19.1)
Farmer	73 (4.2)
Unemployed	36 (2.1)
Others	72 (4.2)
**Education level**	
Illiterate	154 (9.0)
Below SSC/PSC-JSC	237 (13.8)
SSC	267 (15.5)
HSC	309 (18.0)
Undergraduate	464 (27.0)
Graduate or above	286 (16.6)
**Monthly income**	
Below 10,000 BDT	291 (16.9)
10,000–<20,000	174 (10.1)
20,000–<30,000	123 (7.2)
30,000–<40,000	147 (8.5)
40,000–>50,000	67 (3.9)
>50,000	191 (11.1)
Not applicable/Prefer not to say	727 (42.3)
**Residential area**	
Rural	526 (30.6)
Semi urban	278 (16.2)
Urban	916 (53.3)
Media availability **	
TV	1456 (84.7)
Radio	400 (23.3)
Newspaper	795 (46.2)
Internet	1151 (66.9)

* GO-government organization, NGO- Non-government organization; SSC = Secondary school certificate (Year 10); HSC = Higher secondary school certificate (Year 12); PSC = Primary school certificate (Year 5); JSC = Junior School certificate (Year 8). ** Multiple responses were allowed for media availability.

**Table 2:** Knowledge regarding mosquito-borne diseases among the respondents.

Variable	Frequency (%)		
	Yes	No	Uncertain
Have you heard the term “mosquito-borne diseases”?	1679 (97.6)	25 (1.5)	16 (0.9)
Which of the followings are mosquito-borne diseases?			
Dengue	1671 (97.1)	49 (2.8)	0 (0.0)
Chikungunya	1401 (81.4)	319 (18.5)	0 (0.0)
Malaria	1466 (85.2)	254 (14.8)	0 (0.0)
Filaria	918 (53.3)	802 (46.6)	0 (0.0)
The dengue and chikungunya vector bites at daytime	1313 (76.3)	103 (6.0)	304 (17.7)
Malaria vector bites at night-time	1013 (58.9)	96 (5.5)	611 (35.6)
Have you heard the name of the following mosquitoes			
*Aedes* spp.	1565 (90.9)	153 (8.9)	0 (0.0)
*Anopheles* spp.	894 (51.9)	824 (47.9)	0 (0.0)
*Culex* spp.	598 (34.7)	1120 (65.1)	0 (0.0)
Not anyone hear before	94 (5.5)	1327 (77.1)	0 (0.0)
Dengue and chikungunya have specific treatment and medicine	716 (41.6)	506 (29.4)	495 (28.8)
Mosquitoes can be born in both clear and dirty water	1079 (62.7)	429 (24.9)	212 (12.3)
Mosquitoes can breed in stagnant water	1477 (85.8)	41 (2.4)	184 (10.7)
In addition to fever, which of the following are symptoms of Dengue fever?			
Rash	999 (58.0)	721 (42.0)	0 (0.0)
Pain in behind the eyes	849 (49.3)	871 (50.6)	0 (0.0)
Vomiting	981 (57.0)	739 (42.9)	0 (0.0)
Muscle pain	1284 (74.6)	439 (25.3)	0 (0.0)
Do not know	19 (1.1)	1701 (98.8)	0 (0.0)
In addition to fever, which of the following are symptoms of Filariasis?			
Elephantiasis of different organs	1136 (66.0)	584 (33.9)	0 (0.0)
Inguinal or axillary lymphadenopathy	526 (30.6)	1194 (69.4)	0 (0.0)
Skin exfoliation	421 (24.5)	1299 (75.5)	0 (0.0)
Do not know	345 (20.0)	1375 (79.9)	0 (0.0)
In addition to fever, which of the following are symptoms of Malaria?			
Vomiting	960 (55.8)	760 (44.2)	0 (0.0)
Diarrhea	751 (43.6)	969 (56.3)	0 (0.0)
Muscle pain	935 (54.3)	785 (45.6)	0 (0.0)
Do not know	273 (15.9)	1447 (84.1)	0 (0.0)

**Table 3:** Respondent knowledge score about mosquito-borne diseases.

Variables	Frequency (%)
Low (<50)	659 (38.3)
Moderate (50–70)	774 (45.0)
Good (>70+)	287 (16.7)
Overall	60.7 ± 18.1

**Table 4:** Respondent Attitude score about mosquito-borne diseases.

Variables		Frequency (%)
Negative attitudes	Mild (<50)	28 (1.6)
Positive attitudes	Moderate (50–70)	524 (30.4)
	Good (>70+)	1168 (67.9)
Overall		80.4 ± 13.1

**Table 5:** Respondent attitudes towards mosquito-borne diseases.

Variables	Frequency (%)		
	Agree	Disagree	Not Sure
We can prevent mosquito-borne diseases	1625 (94.5)	39 (2.3)	56 (3.3)
Mosquito-borne diseases is a problem in Bangladesh	1673 (97.3)	26 (1.5)	21 (1.2)
Mosquito-borne diseases can be fatal	1604 (93.3)	37 (2.2)	79 (4.6)
We can be affected by mosquito-borne diseases	1667 (96.9)	21 (1.2)	32 (1.9)
More awareness should be generated to prevent mosquito-borne diseases	1670 (97.1)	29 (1.7)	21 (1.2)
Government is taking enough steps to prevent mosquito-borne diseases	783 (45.5)	659 (38.3)	278 (16.2)
Besides government every citizen should work to prevent mosquito-borne diseases	1645 (95.6)	36 (2.1)	39 (2.3)
There is a chance of occurring new types of mosquito-borne diseases in our country	1103 (64.1)	85 (4.9)	532 (30.9)
Which of the following do you think is an obstacle for the prevention of mosquito breeding or mosquito-borne diseases?			
Unawareness	1629 (94.7)	91 (5.3)	0 (0.0)
Financial problem of individual	569 (33.1)	1151 (66.9)	0 (0.0)
Lack of public attachment	897 (52.2)	823 (47.8)	0 (0.0)
Lack of governmental approaches	832 (48.4)	888 (51.6)	0 (0.0)
Do you think your surroundings have mosquito breeding place?	1365 (79.4)	166 (9.7)	182 (10.9)

**Table 6:** Association of sociodemographic factors related to mosquito-borne disease knowledge score among the respondents.

Variables	B	95% Confidence Interval for B		β	t	*P*
		Lower	Upper			
Age	−0.01	−0.06	0.04	−0.01	−0.376	0.707
Gender	−0.07	−0.021	0.06	−0.02	−1.057	0.290
Profession	−0.39	−0.42	−0.35	−0.36	−17.223	<0.001
Monthly Income	0.03	−0.00	0.05	0.03	1.751	0.080
Residential area	0.48	0.40	0.56	0.24	12.086	<0.001
Education level	0.16	0.14	0.18	0.31	15.804	<0.001

R^2^ = 0.41 (*n* = 1720, *p* < 0.001).

**Table 7:** Association of the sociodemographic factors related to mosquito-borne disease attitude score among the respondents.

Variables	B	95% Confidence Interval for B		β	T	*P*
		Lower	Upper			
Age	0.02	−0.03	0.07	0.02	0.786	0.432
Gender	0.07	−0.05	0.19	0.03	1.096	0.273
Profession	−0.06	−0.11	−0.02	−0.08	−3.086	0.002
Monthly Income	0.01	−0.02	0.03	0.01	0.302	0.763
Residential area	0.26	0.18	0.33	0.17	7.020	<0.001
Education level	0.03	0.01	0.04	0.07	2.871	0.004

R^2^ = 0.05 (*n* = 1720, *p* < 0.001).

**Table 8:** Respondents’ prevention practice against mosquito-borne diseases.

Variables	Frequency (%)		
	Yes	Sometimes	Never
Do you kill mosquitoes as you notice them?	1373 (79.8)	324 (18.8)	23 (1.3)
Do you use any of the following to prevent mosquito biting			
Mosquito coil	1475 (85.7)	245 (14.2)	0 (0.0)
Insecticide spray	570 (33.1)	1148 (66.7)	0 (0.0)
Fan	763 (44.3)	957 (55.6)	0 (0.0)
Generating Smokes	316 (18.4)	1403 (81.5)	0 (0.0)
Bed net	1613 (93.7)	107 (6.2)	0 (0.0)
Wear full sleeves dress	271 (15.7)	1449 (84.2)	0 (0.0)
Nets in windows	268 (15.6)	1452 (84.4)	0 (0.0)
Mosquito repellent cream	245 (14.2)	1475 (85.7)	0 (0.0)
Electric bat	723 (42.0)	996 (57.9)	1 (0.1)
Do you keep your surroundings clean?	1308 (76.0)	377 (21.9)	33 (1.9)
Do you contact your local government office/administration if you feel the increase in the number of mosquitoes?	127 (7.4)	331 (19.2)	1259 (73.2)
Do you check and clean water storage in your house such as pot, flowerpot, refrigerator tray, AC?	870 (50.6)	615 (35.7)	233 (13.5)
Do you remove any pots, coconut shell, plastic packets, plastic cups, or anything besides the road that may contain water?	481 (27.9)	778 (45.2)	459 (26.7)
Do seek medical help if you feel feverish?	860 (50.0)	757 (44.0)	102 (5.9)

## Data Availability

All datasets are available upon request to the corresponding authors.
